# Lost Needle During Robot-Assisted Radical Prostatectomy: A Case Report and Literature Review

**DOI:** 10.7759/cureus.42119

**Published:** 2023-07-19

**Authors:** Yohei Koida, Hiroshi Kiuchi, Fumie Yoshioka, Tetsuji Soda, Kenichiro Sekii

**Affiliations:** 1 Urology, Osaka Central Hospital, Osaka, JPN

**Keywords:** lost needle, rarp, management, robotic surgery, laparoscopic surgery

## Abstract

Laparoscopic or robotic radical prostatectomy and partial nephrectomy require needle suturing and manipulation. Although uncommon, if a needle is lost during laparoscopy, locating and removing it is challenging. Here, we report a case of needle loss during robot-assisted laparoscopic radical prostatectomy (RARP). A 51-year-old patient with localized prostate cancer underwent RARP. After vesicourethral anastomosis using a 3-0 Barbed Suture with two threads connected in the tail, the two threads were held with a needle holder. One needle was lost during removal through a 12 mm trocar. A thorough laparoscopic examination of the abdominal cavity identified a needle attached to the abdominal wall, which was successfully removed. Needle loss is uncommon, but familiarity with handling and preventing such cases helps surgeons address further deterioration. Stepwise and intensive exploration should be performed to confirm the needle location.

## Introduction

Laparoscopic or robotic surgery offers significant advantages over open surgery, including less pain, shorter hospital stay, and shorter time to return to society [1,2]. These factors have led to a worldwide increase in the use of laparoscopic surgery. In urological surgery, radical prostatectomies and partial nephrectomies, which require suturing and needle manipulation, are commonly performed laparoscopically. Although very uncommon, finding and removing a lost needle during laparoscopic surgery is challenging. Any foreign objects lost in the abdominal cavity must be removed because leaving them can have serious consequences. Herein, we report a case in which a needle was lost during robot-assisted laparoscopic radical prostatectomy (RARP) and was successfully removed.

## Case presentation

**Figure 1:**
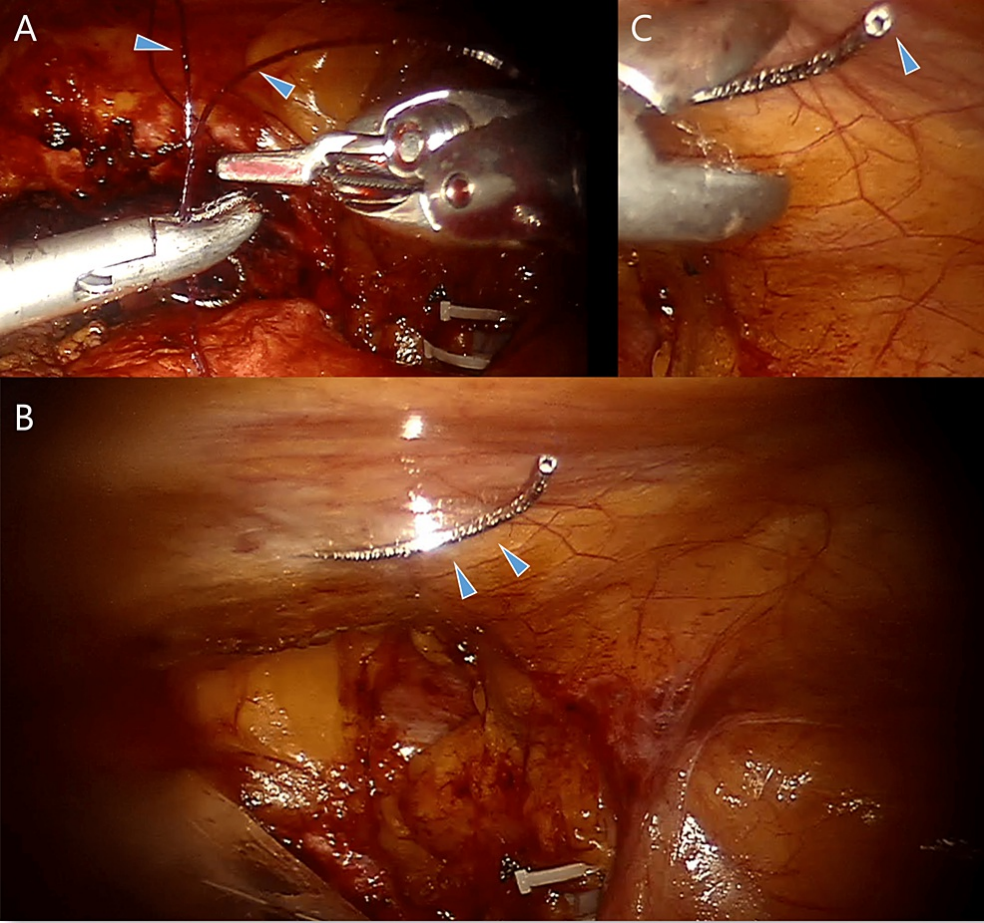
Intraoperative pictures. A: Simultaneous holding of two threads with needles. B: The lost needle is adherent to the abdominal wall. C: The moment of grasping of the lost needle with a needle holder. Arrowhead shows the suture-needle connection from which a thread had been removed.

A 51-year-old man presented with a prostate-specific antigen of 5.07 ng/mL, and a biopsy revealed Gleason 3 + 4 prostatic adenocarcinoma. Metastasis was not detected on a computed tomography scan and bone scintigraphy. The patient was diagnosed with cT2aN0M0 prostate cancer. His weight was 99 kg, height 166 cm, and body mass index (BMI) 33.6 kg/m^2^. The patient underwent RARP via a transperitoneal approach. After the excision of the prostate, the vesicourethral anastomosis was performed using a 3-0 Quill^®^ Barbed Suture with two threads connected in the tail. After the suture was completed, the two threads were cut and held together with a needle holder for removal through a 12 mm trocar (Figure 1A). Threads with and without needles were found after the withdrawal of the needles. First, we recounted the number of needles with the operating room staff; however, one needle was missing. Next, we looked for the needle around the patient, operating room, and cart but could not find it. We considered it highly likely that the needle remained intraperitoneally. A thorough search of the abdominal cavity using da Vinci without moving the patient’s bed was performed; however, we could not find the needle. We removed the da Vinci system and manipulated the camera to search further without moving the bowel. After an approximately 10 min intensive search, we finally found the needle attached to the abdominal wall (Figure 1B). The thread was removed at the needle-suture junction (Figure 1C). Since the needle was only slightly attached to the abdominal wall, it could have been lost again when we tried to remove it. The scopist did not move the camera to avoid losing the needle. The surgeon held the needle holder in each hand and slowly approached the needle to hold it. The moment he touched it, the needle fell off, and he managed to grab it. Finally, the lost needle was successfully removed from the patient. After removing the needle, hemostasis was confirmed, a drain was placed, and the procedure was completed.

## Discussion

Needle loss during abdominal surgery is very rare, with a reported incidence of 0.06-0.11% [3]. Factors that increase the risk of needle loss include a high BMI, the number of laparoscopic instruments used, a surgical team of two or more, equipment failure, unexpected changes during surgery, poor communication among surgical team members, and complex and lengthy laparoscopic procedures [3-5]. In this era of laparoscopic suture procedures, proficiency in dealing with the loss of needles and prevention is considered essential. Some algorithms have been proposed for the management of needle loss [5-7]. First, recount the needles immediately to confirm any missing needles and explore all the operation fields, including outside the abdominal cavity and the operating room floor. Second, the inside of the laparoscopic suction device can accidentally aspirate a needle smaller than the 4-0 needle. Third, visualize the trocar, especially the trocar valves. In most reported cases of needle loss in laparoscopic surgery, the needle was misplaced inside the trocar valve [8]. Fourth, the surgical field is searched using a laparoscope without mobilizing further intra-abdominal structures. It is important to realize that the surgeon must avoid making rapid movements because the movement of the bowel will cause the needle to shift further, making the retrieval process even more troublesome. Finally, imaging studies, x-rays, or computed tomography scans are necessary. The detection rate of x-rays depends on the size of the needle: Needles 4-10 mm, 11-24 mm, and larger than 25 mm have a detection rate of only 29%, 85%, and 99%, respectively [9]. Another report confirmed that needles larger than 19 mm were recognized in 100% of cases, while 13 mm needles were identified in only 13% [10]. Even if the location of the needle was identified by radiography, it is not easy to confirm the actual location of the needle. x-rays of radiopaque objects, such as scissors, are useful.

Needle loss during surgery can have severe consequences, and precautionary measures should be considered. Medina et al. recommended six prevention rules. 1) Hold only one needle at a time. 2) Use needle holders for needle insertion or removal and avoid graspers as substitutes. 3) Proper and clear communication dynamics within the team to ensure a proper final count. 4) Slow needle withdrawal under continuous visualization. 5) Avoid parking needles. 6) Ensure the strength of the suture-needle connection before use [7]. In our case, the two threads with needles were held simultaneously and promptly withdrawn from the trocar without visualization. Holding the threads instead of the needles increased the risk of needle loss. These combinations of these small errors have serious consequences.

Leaving a needle inside the abdominal cavity can have significant medical and legal consequences [7]. Short-term complications have not been reported. The anxiety of the surgeon and the patient may represent a greater problem. Long-term complications included chronic pain, bowel perforation, fistula development, vascular injuries, and recurrent surgery. These adverse events occurred in only 3% of patients [3].

## Conclusions

We present a rare case in which a needle was lost during RARP and was successfully removed. Needle loss is uncommon, but it can have serious consequences. Intensive and stepwise exploration should be performed to confirm the needle location, if required, using imaging techniques. Surgeons must be familiar with the management and prevention of needle loss. The risk should be explained to the patient and his or her family prior to surgery.
